# Analysis of a novel RNA virus in a wild northern white-breasted hedgehog (*Erinaceus roumanicus*)

**DOI:** 10.1007/s00705-019-04414-7

**Published:** 2019-09-23

**Authors:** Gábor Reuter, Éva Várallyay, Dániel Baráth, Gábor Földvári, Sándor Szekeres, Ákos Boros, Beatrix Kapusinszky, Eric Delwart, Péter Pankovics

**Affiliations:** 1 Department of Medical Microbiology and Immunology, Medical Center, University of Pécs, Szigeti út 12, Pecs, 7624 Hungary; 2 Agricultural Biotechnology Institute, National Agricultural Research and Innovation Centre, Gödöllő, Hungary; 3 Evolutionary Systems Research Group MTA Centre for Ecological Research, Tihany, Hungary; 4 Department of Parasitology and Zoology, University of Veterinary Medicine, Budapest, Hungary; 5 Vitalant Research Institute, San Francisco, CA USA; 6 University of California, San Francisco, San Francisco, CA USA

## Abstract

Tombusviruses are generally considered plant viruses. A novel tombus-/carmotetravirus-like RNA virus was identified in a faecal sample and blood and muscle tissues from a wild northern white-breasted hedgehog (*Erinaceus roumanicus*). The complete genome of the virus, called H14-hedgehog/2015/HUN (GenBank accession number MN044446), is 4,118 nucleotides in length with a readthrough stop codon of type/group 1 in ORF1 and lacks a poly(A) tract at the 3′ end. The predicted ORF1-RT (RdRp) and the capsid proteins had low (31-33%) amino acid sequence identity to unclassified tombus-/noda-like viruses (Hubei tombus-like virus 12 and Beihai noda-like virus 10), respectively, discovered recently in invertebrate animals. An *in vivo* experimental plant inoculation study showed that an *in vitro*-transcribed H14-hedgehog/2015/HUN viral RNA did not replicate in *Nicotiana benthamiana*, *Chenopodium quinoa*, or *Chenopodium murale*, the most susceptible hosts for plant-origin tombusviruses.

Recently, the discovery of novel viruses has been dramatically enhanced by the use of culture-free viral metagenomics and next-generation sequencing techniques [1]. However, in many cases, this approach does not allow the host species of the novel virus to be identified. In addition to the host origin, dietary sources or other forms of contamination are also possible sources of certain viruses, especially in faecal specimens. The picture is complicated by the fact that some viruses are traditionally thought to have a narrow host range but might actually have a much wider host range [2].

For example, a recent study investigating the RNA virome of invertebrate animals identified 1,445 novel viruses by viral metagenomics, including many tombus- and tombus-like viruses [3]. Tombusviruses (family *Tombusviridae*) are positive-sense, single-stranded RNA viruses (genome length, 3.7 to 4.8 kb) that are currently thought to be plant viruses with relatively narrow host range [4]. The origin and pathogenic roles (if any) of tombus-like viruses in invertebrate specimens are presently uncertain. On the other hand, a relative of the tombusviruses, Providence virus (the only member of the family *Carmotetraviridae*), a positive-sense, single-stranded RNA virus, is thought to be an insect virus [5, 6] that replicates in lepidopteran species (*Helicoverpa zea*), although a recent study indicated that it was also able to replicate in the human HeLa cell line [7, 8]. These data show that the possible full spectrum of host and target cells infected by members of some viral families may be wider than currently appreciated.

The northern white-breasted hedgehog (*Erinaceus roumanicus*) is a medium-sized mammal belonging to the genus *Erinaceus,* family Erinaceidae (Mammalia: Eulipotyphla). It can be found in Central Europe, the Balkan Peninsula, the Adriatic Islands, Ukraine and Russia, extending to the Ob River in Siberia. These animals inhabit farmland, parks and gardens in rural and urban areas, scrubby habitats at the edge of forests, and shrubby vegetation [9]. They feed on earthworms, insects (larvae, pupae and imagos), snails, slugs, small vertebrates (amphibians, lizards and occasionally young rodents), chicks, bird eggs, and even some berries and fruits.

Here, we report the complete genome sequence of a novel positive-sense, single-stranded RNA virus from a wild northern white-breasted hedgehog; however, the host origin of the studied virus could not be determined in spite of *in vivo* experimental inoculation studies with the most susceptible plants for tombusviruses.

A total of 20 faecal specimens were collected from northern white-breasted hedgehogs [10]. Eight faecal samples (H1, H2, H4, H9-H11, H13 and H14) were collected between April and June in 2015 from carcasses of road-killed hedgehogs from urban environments, seven of which were from streets in the capital, Budapest, and one was from the suburb of Szentendre, Hungary. Twelve faecal samples (MR1-MR9 and MR11-MR13) were collected from wild-living hedgehogs collected from April to July in 2015, from a natural wild area near the town of Pécs, Baranya County, Southwest Hungary. H10 and H11 were juvenile animals (< 1 year old), and the others were adults (> 1 year old), as judged based on their size and – when available – skull bone ossification. Samples were collected by qualified biologists with valid permission from the National Inspectorate for Environment, Nature and Water (4018-4/2015). A specimen pool containing three faecal samples (H9, H13 and H14) was randomly selected for viral metagenomics analysis [10]. Briefly, the specimen was diluted in PBS, passed through a 0.45-μm sterile filter (Millipore), and centrifuged at 6,000 × *g* for 5 min. The filtrate was then treated with a mixture of DNases and RNases to digest unprotected nucleic acids [11]. Viral-particle-protected nucleic acids were extracted using the QIAamp spin-column technique (QIAGEN) and subjected to a viral metagenomic analysis using sequence-independent random amplification [12]. A viral cDNA library was constructed using a Nextera XT DNA Library Preparation Kit (Illumina) sequenced on a HiSeq Illumina platform according to the manufacturer’s instructions as described previously [11]. The reads were then trimmed; assembled *de novo,* and analyzed using an in-house pipeline [11]. The reads and contigs greater than 100 bp were compared to the GenBank protein database (BLASTx). Virus-family-level categorization of viral metagenomic reads was based on the best BLASTx-scores (E-value ≤ 10^−10^). Sequence-specific screening primer pairs (SCR-F, 5′-AATTCAATTACCTACTGGCCTCAT-3′, corresponding to nt positions 2169-2192 and SCR-R: 5′, TCCTAGCGCGGTGTTCATGTCTCC-3′, corresponding to nt positions 2536-2513 of the study strain) were designed based on the RNA-dependent RNA polymerase (RdRp) sequence contig to identify the specific viral RNA from the specimen pool. Different sets of specific primers were designed based on the metagenomic reads and contigs to obtain the complete viral genome sequence (H14-hedgehog/2015/HUN) and for verification of the full-length metagenomic contigs by the primer-walking [13] and 5′/3′RACE methods [14]. Primer sequences are available upon request. PCR products were sequenced directly using an automated sequencer (ABI PRISM 310 Genetic Analyzer, Applied Biosystems, Stafford, USA). All faecal specimens (N = 20) from northern white-breasted hedgehogs were tested by RT-PCR using the SCR-F/R primers. In addition, available coagulated blood, ear skin, abdominal muscle, spleen and liver tissues collected from animal H14 were also tested by RT-PCR using SCR-F/R primers.

Coding regions were identified using NCBI Open Reading Frame Finder (https://www.ncbi.nlm.nih.gov/orffinder/). All evolutionary analyses were conducted in MEGA 6.06 [15]. The non-structural (RNA-dependent RNA polymerase) amino acid (aa) sequences of the study strain and the representative reference viruses were aligned using MEGA 6.06 and pre-tested using a best amino acid model (ML) search. Dendrograms were constructed by the maximum-likelihood method based on the Poisson model with gamma distribution (+G) and invariable sites (+I). To assess the reliability of the trees, 1000 replicates of bootstrap analysis were performed.

The genomic DNA of H14-hedgehog/2015/HUN was cloned for *in vitro* transcription and *in vivo* plant inoculation studies. Briefly, primers were designed based on the sequence of the putative H14-hedgehog/2015/HUN (H14_HedgehogT7_1F, TAATACGACTCACTATA**GGGTACATTATGGCCCAAAATGATG**; H14_Hedgehog_4118RXbaI, TCTCTAGACTCGA**GCACTTATCTTGTACTAGGTTATC**; bold letters represent sequences from the virus under study). cDNA was synthesized using a RevertAid First Strand cDNA Kit (Thermo Fisher Scientific) according to the manufacturer’s instructions from RNA extracted from hedgehog faeces using the primer H14_Hedgehog_4118RXbaI. The virus-specific cDNA was then used as a template for PCR amplification with Q5 High-Fidelity DNA Polymerase (New England Biolabs) (initial denaturation for 30 s at 98 °C, 40 cycles of denaturation for 10 s at 98 °C, annealing for 30 s at 70 °C, extension for 3 min at 72 °C, and a final extension for 2 min at 72 °C) using the primers H14_HedgehogT7_1F and H14_Hedgehog_4118RXbaI. The PCR-product was isolated from a 1.2% agarose gel using a GeneJet Gel Extraction Kit (Thermo Fisher Scientific), and it was then ligated into pJET1.2/blunt Cloning Vector (CloneJET PCR Cloning Kit, Thermo Fisher Scientific), introduced into *E. coli* (DH5alpha) by transformation. The plasmid DNA was purified from an overnight bacterial culture using a Nucleospin Plasmid Purification Kit (Macherey Nagel). Using restriction endonuclease sites for NotI (site on the pJET1.2 vector) and XbaI, the entire virus genome was excised from pJET1.2 and then cloned into a pUC18 vector digested with SmaI and XbaI (Thermo Fisher Scientific). The recombinant plasmid was then linearized with XbaI, treated with proteinase K, purified by phenol/chloroform extraction, and precipitated with ethanol. For *in vitro* transcription we used this linearized plasmid as a template in a 50-μl reaction mixture containing 10 μl of 5X reaction buffer for T7 RNA polymerase (Thermo Fisher Scientific) 1 μl of RiboLock RNase Inhibitor (40 U/μl, Thermo Fisher Scientific), 5 μl of an NTP mixture (20 mM, Thermo Fisher Scientific), and 1.5 μl of T7 RNA polymerase (15 U/μl, Thermo Fisher Scientific). The presence of the transcript was confirmed by electrophoresis of an aliquot on a 1.2% agarose gel.

During the plant inoculation study, *Nicotiana benthamiana*, *Chenopodium quinoa* and *C. murale* plants were grown at 21-22 °C. Eight- to ten-week-old plants with four leaves were leaf-rub inoculated with transcripts containing or not containing (mock) inoculation buffer [16]. Infected plants were kept at 21-22 °C in a growth chamber with a 16-h light/8-h dark cycle. For RNA analysis, total nucleic acid was extracted from inoculated and systemic leaves of *N. benthamiana* plants at 1 and 6 hours, 1 and 10 days and 1, 2, and 3 weeks post-inoculation, in the case of *C. quinoa* and *C. murale*, RNA was extracted at 1 day and 1, 2 and 3 weeks post-inoculation. Frozen plant materials were homogenized in an ice-cold mortar, suspended in 650 µl of extraction buffer (100 mM glycine, pH 9.0, 100 mM NaCl, 10 mM EDTA, 2% SDS, and 1% sodium lauroyl sarcosinate), mixed with an equal volume of phenol, and centrifuged for 5 minutes. The aqueous phase was treated with an equal volume of phenol and chloroform:isoamyl-alcohol, and after subsequent treatment with chloroform:isoamyl-alcohol, it was precipitated with ethanol and suspended in sterile water. For Northern blot analysis, total nucleic acid was separated on a 1.2% TBE agarose gel and blotted onto Amersham Hybond-NX membrane (GE Healthcare) by the capillary method using 20x SSC (3 M NaCl and 0.3 M Na-citrate, pH 7.0) [17]. Virus-specific, ^32^P-labeled DNA probes were prepared using a DecaLabel DNA Labeling Kit (Thermo Fischer Scientific), using PCR-amplified and purified product of the cloned H14-hedgehog/2015/HUN as a template. Hybridization was carried out overnight at 65 °C in Church buffer (0.5 M sodium phosphate buffer, pH 7.2, containing 1% bovine serum albumin, 1 mM EDTA, 7% SDS) with the appropriate radioactively labeled probe, then washed for 5 min in 2X SSC with 0.1% SDS and for 15 min in 0.5X SSC with 0.1% SDS at the temperature of the hybridization and exposed to an X-ray film.

A specimen pool containing three faecal samples from northern white-breasted hedgehogs was subjected to viral metagenomics analysis. After *de novo* assembly of 23,004,466 reads, 315,105 sequence reads were obtained showing translated protein similarity to viral sequences from this sample pool. The detected sequences containing more than 50 reads were from viruses of the families *Picornaviridae* (N = 151,949, including dicipivirus) [10], unclassified viruses (N = 83,300), *Tombusviridae* (N = 54,383), *Virgaviridae* (N = 10,829), *Microviridae* (N = 8,810), *Alphaflexiviridae* (N = 2,250), *Circoviridae* (N = 1,536), *Permutotetraviridae* (N = 441), *Luteoviridae* (N = 398), *Parvoviridae* (N = 370), *Podoviridae* (N = 225), *Tymoviridae* (N = 99), *Carmotetraviridae* (N = 79), *Iflaviridae* (N = 71), and *Dicistroviridae* (N = 67). The reads including the five contigs corresponding to members of the family *Tombusviridae* were pre-selected for *in silico* sequence analysis. Tombusviral reads (N = 36,886) including three contigs (642, 1,194 and 2,132 nucleotides in length) were related to carnation ringspot virus of the genus *Dianthovirus,* with more than 99% aa sequence identity. Two groups of reads (11,174 and 3,353 reads) were related to tobacco necrosis virus D of the genus *Betanecrovirus* and tobacco necrosis virus A of the genus *Alphanecrovirus*, respectively. Five reads including a contig (2,095 nt in length) were related to carrot mottle virus of the genus *Umbravirus*. Sixty-seven reads including a contig (3,732 nt in length) were related to Sclerotinia sclerotiorum umbra-like virus 1 of the genus *Umbravirus*. The remaining 2,898 tombusvirid-like reads were related to aureusviruses, alphacarmoviruses, betacarmoviruses, gammacarmoviruses, machlomoviruses, panicoviruses and zeaviruses. The longest contig (3,732 nt in length) was selected for further analysis. Using the screening primer-pairs (SCR-F/R), one (H14) of the three specimens of the sample pool were RT-PCR positive for the study sequence. To characterize the complete viral RNA genome from sample H14, different sets of specific primers were designed on the basis of the metagenomic sequence contig. Amplicons were sequenced directly by the Sanger method. The complete genome of the virus strain H14-hedgehog/2015/HUN (GenBank accession number MN044446) is 4,118 nt long without a poly(A) tract at the 3′ end (Fig. 1). The G + C content of the genome is 50.6%. Five potential ORFs with (+) orientation were identified that encoded putative proteins longer than 100 aa (Fig. 1). Only two (ORF1-RT and ORF2; Fig. 1) of the five ORFs had any sequence match in the GenBank database. ORF1-RT (533 aa/1,602 nt, including the RdRp) had 31% and 32% aa sequence identity to the non-structural proteins of an unclassified virus, Hubei tombus-like virus 12 (NC_032853; E-value, 8 × 10^−48^; query coverage 81%) from freshwater shellfish [3] and Providence virus (NC_014126; E-value 10^−46^; query coverage 77%), the sole member of the family *Carmotetraviridae*, respectively. The ORF1-RT (RdRp) encodes the seven (I-VII) canonical domains, including the GDD polymerase core aa motif [6, 18] (Fig. 1). Nucleotide sequence analysis and the context of the stop codon (U_1568_AGCAACUA) of the 516-aa-long potential ORF1 sequence at the 5’ end in the second reading frame correspond to the consensus sequence for a readthrough stop codon (UAGCARYYA) of type/group 1[19] which is characteristic of plant viruses [20] (Fig. 1). This means that the ORF1-RT (RdRp) could be translated through suppression of termination (stop codon readthrough) at the end of ORF1 [3; https://viralzone.expasy.org/859?outline=all_by_protein]. ORF2 (340 aa/1,023 nt) had 33% aa sequence identity (E-value, 4 × 10^−15^; query coverage, 38%) to the capsid protein of the unclassified Beihai noda-like virus 10 (KX883117) from tiger crab [3]. ORF2 is potentially translated from a subgenomic RNA [6]. The H14-hedgehog/2015/HUN genome does not encode the ribosome skipping motif (NPG↓P) present in Providence virus [6].

**Fig. 1:**
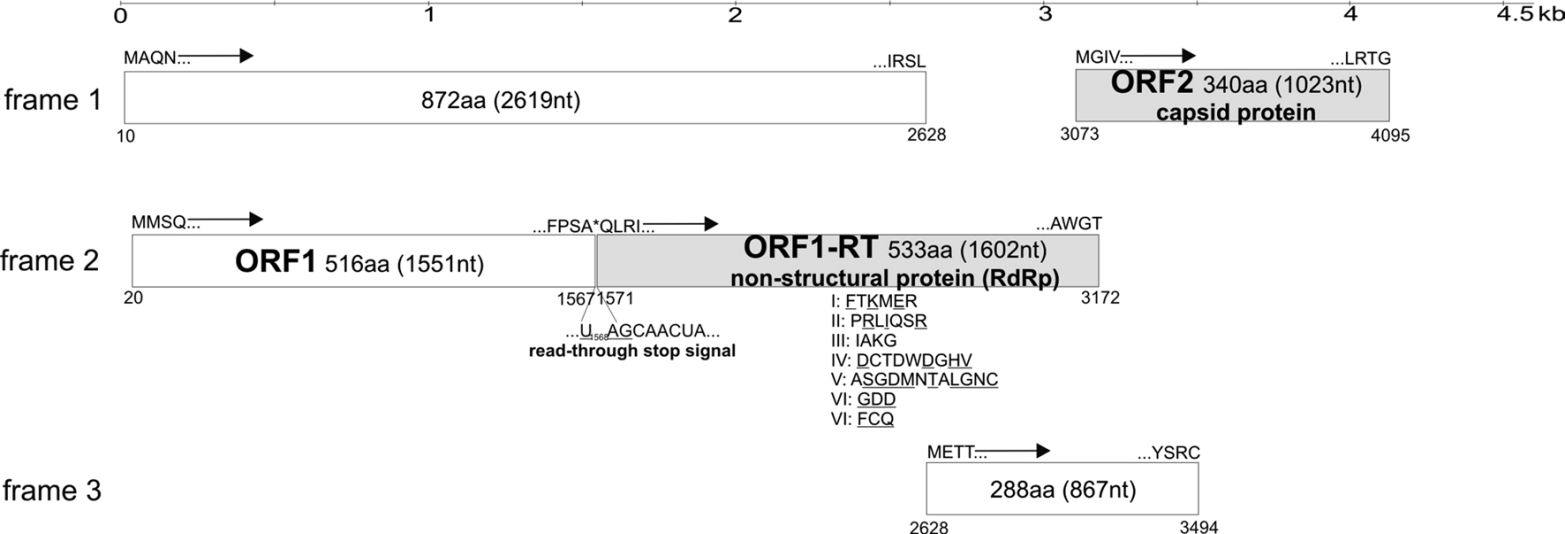
Schematic genome map of the virus strain H14-hedgehog/2015/HUN (GenBank accession number MN044446). Five potential ORFs with (+) orientation were identified that encoded putative proteins longer than 100 aa. Nucleotide (nt) and amino acid (aa) lengths are indicated in each gene box. Each predicted ORF is drawn to scale. The first and last nt positions (Arabic numbers) and the first and last four aa sequences of the given ORF are shown at the borders of each cassette. Arrows indicate the transcription direction of the potential ORFs in the 1^st^, 2^nd^ and 3^rd^ reading frame. The two ORFs (ORF1-RT and ORF2) that 5 had aa sequence matches in GenBank are indicated in grey. In reading frame 2, the ORF1-RT RNA-dependent RNA polymerase (RdRp) sequence is translated through a potential alternative, suppression-of-termination or stop codon readthrough mechanism to produce a full-length protein with an extended C-terminus of ORF1 [6]. The UAG stop codon at nt 1568-1570 that is part of the readthrough stop signal between nt 1568 and 1576 is indicated by an asterisk. The ORF1 encodes the seven canonical RdRp domains [6, 18] (conserved amino acids are underlined)

Phylogenetic analysis based on the nonstructural protein (RdRp) sequence of ORF1-RT showed the distant phylogenetic position of H14-hedgehog/2015/HUN from the representative members of the family *Tombusviridae* and Providence virus (family *Carmotetraviridae*) (Fig. 2).

**Fig. 2:**
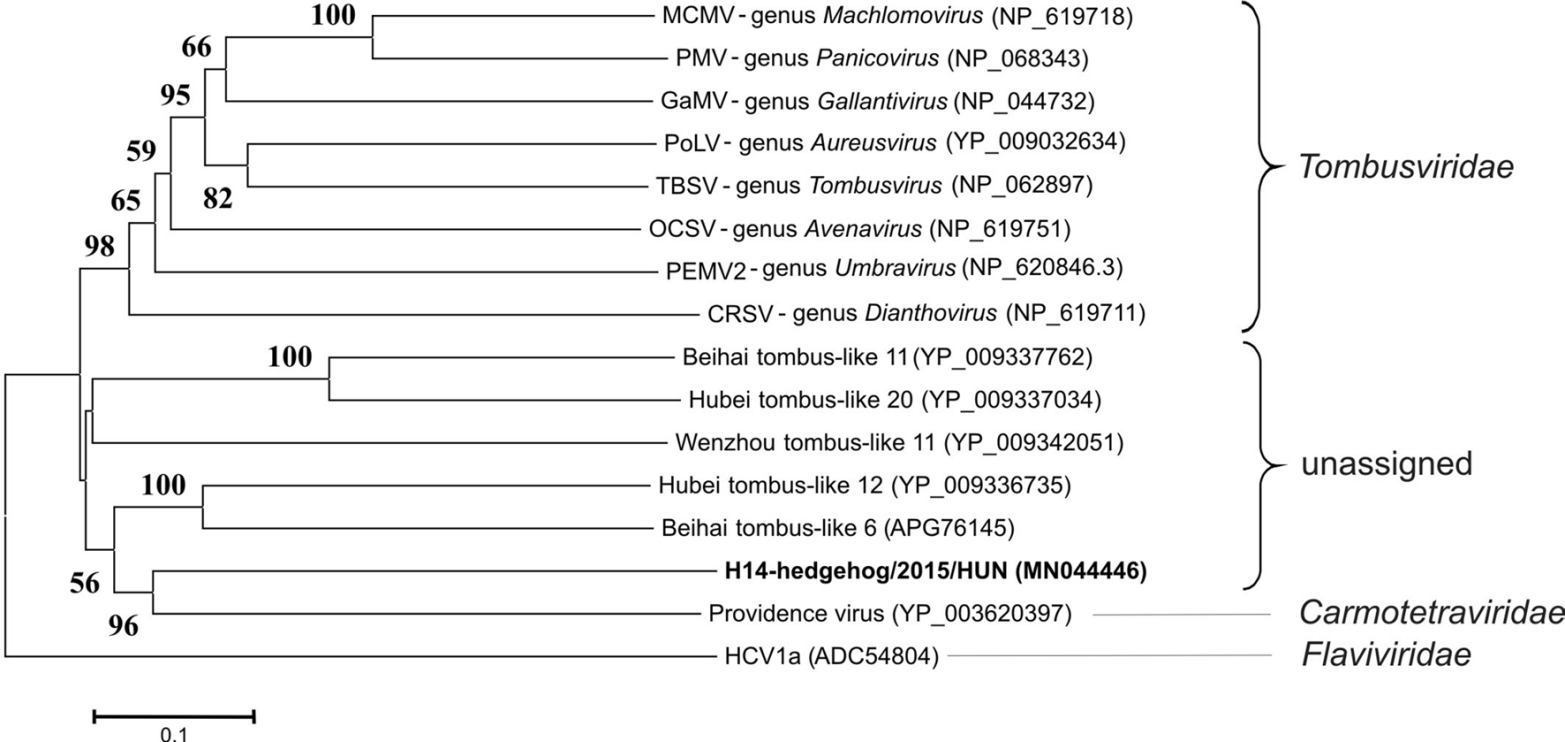
Phylogenetic analysis of virus strain H14-hedgehog/2015/HUN (GenBank accession number MN044446) (bold letters), representative tombusviruses (family *Tombusviridae*) and Providence virus (family *Carmotetraviridae*) based on the predicted protein sequence of the RNA-dependent RNA polymerase encoded by ORF1-RT. Recently reported unassigned tombusvirus-like viruses from invertebrates [3] were also included. The evolutionary analysis was conducted in MEGA6.06 [15]. Bootstrap values were determined using 1000 replicates. The tree is drawn to scale, with branch lengths representing the number of substitutions per site. Hepatitis C virus (HCV) was used as an outgroup. Abbreviations: MCMV, maize chlorotic mottle virus; PMV, panicum mosaic virus; GaMV, galinsoga mosaic virus; PoLV, pothos latent virus; TBSV, tomato bushy stunt virus; OCSV, oat chlorotic stunt virus; PEMV2, pea enation mosaic virus 2; CRSV, carnation ringspot virus

Based on these results we compared the complete genome sequence of H14-hedgehog/2015/HUN with the viral metagenomic reads (N = 79) matching Providence virus and found that they were completely identical to the corresponding canonical domain IV of the RdRp region of H14-hedgehog/2015/HUN (Fig. 1) but had low sequence similarity to the corresponding region of Providence virus.

For investigation (confirmation or exclusion) of the potential plant origin of strain H14-hedgehog/2015/HUN, an *in vitro* transcript (named TR1) of the cloned H14-hedgehog/2015/HUN was used to inoculate *N. benthamiana* plants. Inoculated leaves were tested for the presence of the virus at 1 and 6 hours post-inoculation (hpi) and 10 and 24 days post-inoculation (dpi) by Northern blot using the virus-specific radioactively labeled probe. The strongest signal was detected at 1 hpi, and its intensity decreased steadily, reaching zero at 24 dpi. Although a signal could be detected after 10 dpi, it disappeared later, showing that it was only the signal of the remaining RNA from the initial transcript rather than a replicated virus (Fig. 3A).

**Fig. 3:**
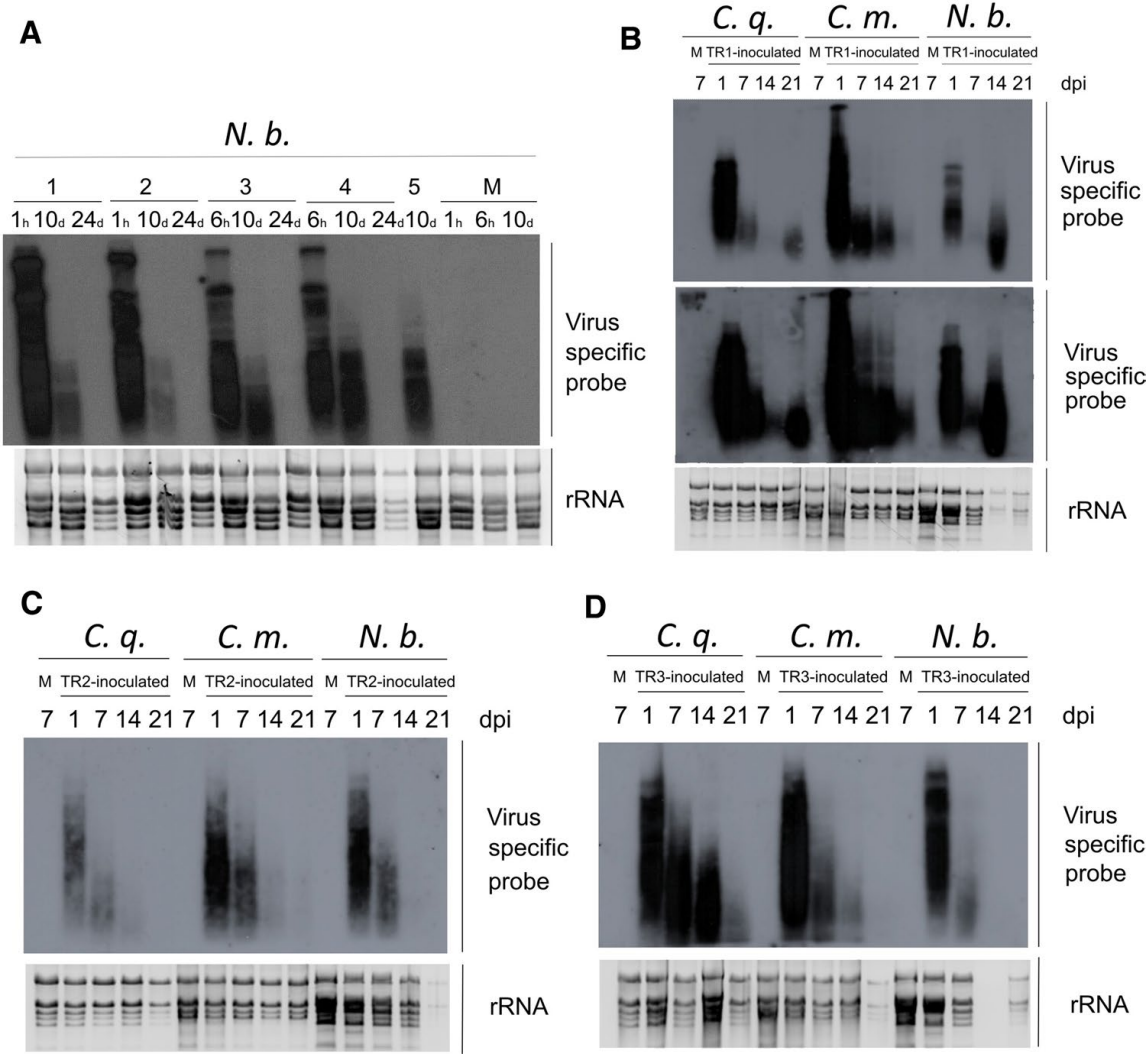
(**A**) Investigation of possible replication of H14-hedgehog/2015/HUN (GenBank accession number MN044446) in *N. benthamiana.* Leaves of five (1-5) 8- to 10-week-old *N. benthamiana* plants were leaf-rub inoculated with an *in vitro* transcript TR1 produced from a clone of H14-hedgehog/2015/HUN. RNA was extracted from the inoculated leaves at 1 hour (h) and 6 hours (h), 10 days (d) and 24 days (d) post-inoculation. Mock-inoculated plants (M) were inoculated with a buffer containing no transcript. RNA was separated on a 1.2% agarose gel, blotted onto a Nytran NX membrane, and hybridized with a virus-specific radioactively labeled probe. rRNA on an ethidium-bromide-stained gel served as a loading control, and it is shown as the negative of the UV light photo. (B, C, and D) Investigation of possible replication of H14-hedgehog/2015/HUN (MN044446) in *C. quinoa* (*C. q.*), *C. murale* (*C. m.*) and *N. benthamiana* (*N. b.*). Leaves of four 8-to 10-week-old test plants were leaf-rub inoculated with three individual *in vitro* transcripts: (**B**) TR1, (**C**) TR2 and (**D**) TR3, produced from the clones of H14-hedgehog/2015/HUN. RNA was extracted from the inoculated leaves at 1, 7, 14, and 21 days post-inoculation (dpi). Mock-inoculated plants (M) were inoculated with a buffer containing no transcript. Samples from mock-inoculated plants were collected at 7 dpi. RNA was separated on a 1.2% agarose gel, blotted onto a Nytran NX membrane, and hybridized with a virus-specific radioactively labeled probe. rRNA on an ethidium-bromide-stained gel served as a loading control. The upper panel in **B** shows a shorter time, while the middle panel shows a longer exposure time

To exclude any possible unwanted mutations occurring during bacterial multiplication of the recombinant virus that might destroy its infectivity, three individual clones (TR1, TR2 and TR3) were used as a template for *in vitro* transcription. The infectivity of these transcripts was tested on *N. benthamiana* and two additional herbaceous plants, *C. quinoa* and *C. murale*. Results of these infection experiments showed the same trend: the signal in the inoculated leaves decreased with time, indicating that neither of the transcripts could replicate in either plant (Fig. 3B-D).

Using the SCR-F/R screening primer pairs, only the faecal specimen collected from animal H14 was RT-PCR positive for the study virus (1/20 faecal samples, 5%). Available tissue samples collected from hedgehogs H14 were also tested for H14-hedgehog/2015/HUN virus by RT-PCR using the SCR-F/R primers. Two (blood and muscle tissue) of the five tissue samples were RT-PCR positive, and both were confirmed to contain H14-hedgehog/2015/HUN virus sequences by direct nucleotide sequencing. Sequence comparisons showed 100% nucleotide sequence identity within the amplified 368-bp-long RdRp region between the H14 sequences originating from faeces, blood and muscle.

A large number and genetically diverse viruses were detected by viral metagenomics and next-generation sequencing methods from faecal samples from a wild northern white-breasted hedgehog. Collectively, these viruses have a wide known (and for some, unknown) host range, including bacterial, plant, fungal, insect and vertebrate hosts. In addition, numerous genetically diverse tombus- and tombus-like viruses were also identified in these faecal samples. After the characterization of a novel dicipivirus from these specimens [10], this study reports the genetic analysis of a novel virus whose tropism was further tested *in vivo* by experimental inoculation of plants with viral RNA.

H14-hedgehog/2015/HUN shows high genetic divergence in ORF-RT relative to its closest known relative in the family *Tombusviridae*. On the other hand, the genome organization of H14-hedgehog/2015/HUN has features similar to those of Providence virus in the family *Carmotetraviridae* [6]. Based on the similar genome organization of H14-hedgehog/2015/HUN and Providence virus [6], the ORF1, ORF1-RT, ORF2 and 872-aa-long ORF of unknown function in reading frame 1 of H14-hedgehog/2015/HUN genome are all potentially active. Phylogenetic analysis of the viral RdRp domain showed that H14-hedgehog/2015/HUN clusters together with an unclassified group of tombus-like viruses from invertebrate animals in a “Tombus-Noda clade” that was described recently (during our study) by Shi et al. [3]. Whether these viruses represent a new viral genus within a known virus family or a new viral family will require sequencing and phylogenetic analysis of additional related strains.

Initially, because of the potential dietary origin of this genome in hedgehog faeces, we looked for a potential plant host for H14-hedgehog/2015/HUN. To investigate the replication potential of H14-hedgehog/2015/HUN in plants, we used *in vivo* experimental plant RNA inoculation. We could not demonstrate replication of H14-hedgehog/2015/HUN virus in *N. benthamiana*, *C. quinoa*, or *C. murale*, the most susceptible plant systems for tombusvirus replication [4, 21]. We therefore could not demonstrate that H14-hedgehog/2015/HUN is a plant-infecting virus. Further tests including other plant hosts, including monocots, could help to clarify this issue. Interestingly, H14-hedgehog/2015/HUN was identified not only in the faecal sample of the infected hedgehog but also extraintestinally, in its blood and muscle tissue. Further investigations are needed to determine whether the extraintestinal presence of the viral RNA is a result of contamination, diffusion/transport from the gut, or active replication in hedgehog cells, resulting in viremia. This last possibility is reinforced by a recent study showing that Providence virus is able to replicate in a mammalian, human cell culture line (HeLa) [7, 8].

In summary, we have described a novel RNA virus from faecal, blood and muscle samples of a vertebrate animal that initially exhibited a distant relationship to plant viruses and viruses detected in invertebrates. Based on the results of this study, plant tropism could not be detected for H14-hedgehog/2015/HUN. Further investigations are needed to determine whether this genome is of dietary origin and does not replicate in hedgehog cells or is capable of replication in either vertebrate or invertebrate (e.g., insect) cells.
